# The Human Coronavirus Disease COVID-19: Its Origin, Characteristics, and Insights into Potential Drugs and Its Mechanisms

**DOI:** 10.3390/pathogens9050331

**Published:** 2020-04-29

**Authors:** Lo’ai Alanagreh, Foad Alzoughool, Manar Atoum

**Affiliations:** Department of Medical Laboratory Sciences, Faculty of Applied Medical Sciences, The Hashemite University, Zarqa 13133, Jordan

**Keywords:** COVID-19, SARS-CoV-2, antiviral therapies

## Abstract

The emerging coronavirus disease (COVID-19) swept across the world, affecting more than 200 countries and territories. Genomic analysis suggests that the COVID-19 virus originated in bats and transmitted to humans through unknown intermediate hosts in the Wuhan seafood market, China, in December of 2019. This virus belongs to the *Betacoronavirus* group, the same group of the 2003 severe acute respiratory syndrome coronavirus (SARS-CoV), and for the similarity, it was named SARS-CoV-2. Given the lack of registered clinical therapies or vaccines, many physicians and scientists are investigating previously used clinical drugs for COVID-19 treatment. In this review, we aim to provide an overview of the CoVs origin, pathogenicity, and genomic structure, with a focus on SARS-CoV-2. Besides, we summarize the recently investigated drugs that constitute an option for COVID-19 treatment.

## 1. Introduction

The coronavirus disease 2019 (COVID-19) was first identified in patients with severe respiratory disease in Wuhan, China. The causative agent was a novel coronavirus scientifically named severe acute respiratory syndrome coronavirus 2 (SARS-CoV-2) [1,2]. Since its discovery, more than 2,850,000 cases have been infected, including nearly 200,000 who have died. The worrisome features of COVID-19 are its apparent ability to spread readily and its propensity to cause severe disease in older adults and patients with existing health conditions [3,4]. Moreover, coronaviruses are well-known to mutate and recombine [5]. The genomic sequence of SARS-CoV-2 has changed since it was first reported. Some scientists believe that these changes have enhanced the virulence of the virus and there are two circulating strains, deadly strain “L”, and less virulent one “S” [6]. Currently, there are no specific antiviral treatments or vaccines available for COVID-19. Treatments are mainly focusing on symptomatic and respiratory support according to protocols issued by the health authority in each country, where many countries are following WHO protocol [3,4]. Nearly all cases with severe symptoms accept oxygen. In critical cases and life-threatening situations, passive immunization through the convalescent plasma and immunoglobulin G transfusion can be used as a rescue treatment [7]. Given the current situation, finding a treatment has become a global public health priority. Many companies are working on finding a medication or a viable vaccine for SARS-CoV-2. However, this is not a short process; scientists estimate the period to have one, about 12–18 months.

The COVID-19 is the third novel coronavirus to cause a large-scale epidemic in the twenty-first century after the Severe Acute Respiratory Syndrome Coronavirus (SARS-CoV) in 2003 [8,9,10] and the Middle East Respiratory Syndrome Coronavirus (MERS-CoV) in 2012 [11,12,13]. Our knowledge and experience in fighting the previous two epidemics can be used for creating treatment strategies against this pandemic. Recently, different medications and a combination of drugs have shown promising results. For example, the combination of chloroquine and azithromycin [14]. Furthermore, the antiviral drug remdesivir and the malaria drug chloroquine have gained much attention globally as potential medications for COVID-19 treatment. On the other hand, different companies are working on finding a treatment in a creative method, antiviral RNA, for example [15].

Given the lack of specific treatment for COVID-19, many groups are working urgently to find an alternative strategy to control the replication and spreading of the virus. We have explored different scientific databases searching for the most promising options for the treatment of COVID-19 and provide an overview of our findings. We are going to review the characteristics of coronaviruses, its origin, genome structure, and replication to explain how each treatment strategy could act on preventing or slowing the viral infection.

## 2. Coronaviruses

Coronaviruses (CoVs) are a large group of viruses common among many animals, including humans. They can cause respiratory illnesses in humans and gastrointestinal illnesses in animals. Under the electron microscope, virions of CoVs have large peplomers that make it look like a crown, hence the name corona, meaning “crown” or “halo” [16,17,18]. Before 2003, human CoVs were not considered a deadly virus. The circulating strains were causing mild symptoms in immunocompetent people. Typically, coronavirus symptoms include runny nose, cough, sore throat, headache, and fever that can last for several days. However, in immunocompromised patients, there is a chance that the virus could cause a lower respiratory illness like pneumonia and bronchitis [16,17]. In 2003, the world was shocked by the first pandemic of the 21st century; the Severe Acute Respiratory Syndrome Coronavirus (SARS-CoV) emerged in Guangdong, China, resulting in 774 deaths and more than 8000 patients [16,19,20]. Nine years later, a strain of CoV evolved in Saudi Arabia to cause the Middle East Respiratory Syndrome Coronavirus (MERS-CoV), approximately 2500 cases have been confirmed, including 861 deaths with a fearful case–fatality rate of 34.4% [20].

### 2.1. Coronaviruses Diversity

CoVs members belong to the subfamily *Coronovirinae* within the family *Coronaviridae* and the order *Nidovirales*. Based on their protein sequences and phylogenetic relationships, members of the *Coronavirinae* subfamily can be classified into four groups, *Alphacoronaviruses*, *Betacoronaviruses*, *Gammacoronaviruses*, and *Deltacoronaviruses*. *Gammacoronaviruses* and *Deltacoronaviruses* infect birds and might infect mammals, but never reported to cause any illnesses in humans [20,21]. On the other hand, *Alphacoronaviruses* and *Betacoronaviruses* are capable of causing respiratory illnesses in humans and gastrointestinal illnesses in animals. Before December 2019, six common coronaviruses (members of *Alphacoronaviruses* and *Betacoronaviruses*) were known to infect humans. HCoV-229E, and HCoV-NL63 and they belong to the Alphacoronaviruses. HCoV-OC43, and HCoV-HKU1 belong to lineage A *Betacoronaviruses*, and the two deadly viruses SARS-CoV and MERS-CoV belong to lineages B and C of *Betacoronaviruses*, respectively [20]. The genomic analysis found that SARS-CoV-2 belongs to the *Betacoronavirus* group, lineage B [4,5].

CoVs are zoonotic pathogens originating in animals and can be transmitted to humans through direct contact. All CoVs that caused epidemics (including COVID-19) are believed to be originated in bats. Bats are hosts of many coronaviruses [17,22]. However, in most cases, these viruses were transmitted to humans through an intermediate animal host. SARS-CoV started through direct contact with market civets cats [23]. MERS-CoV transmitted directly to humans from dromedary camels [11,12,13]. The COVID-19 is suspected to be emerged in the seafood market in Wuhan, China, [1,20]. Most of the early reported cases have been in that market, which was closed later by the Chinese authority. Evolutionary analysis of COVID-19 virus revealed that it is most similar to the bat SARS-like coronaviruses, and for the similarity, it was named SARS-CoV-2. In summary, most of the scientific report believes that SARS-CoV-2 was originated in bats and transmitted to humans through intermediate animal host in the seafood market [5]. Nevertheless, researchers are yet to find a definitive answer to which animal serves as an intermediate host.

### 2.2. Coronavirus Genome Structure and Replication

**Figure 1:**
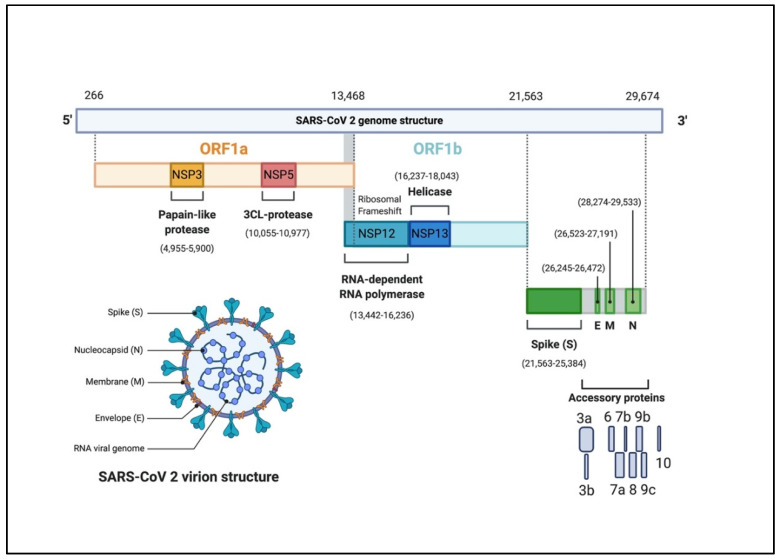
The genomic organization of SARS-CoV-2. The genome encodes two large genes ORF1a (yellow), ORF1b (blue), which encode 16 non-structural proteins (NSP1– NSP16). These NSPs are processed to form a replication–transcription complex (RTC) that is involved in genome transcription and replication. For example, NSP3 and NSP5 encode for Papain-like protease (PLP) and 3CL-protease, respectively. Both proteins function in polypeptides cleaving and block the host innate immune response. NSP12 encodes for RNA-dependent RNA polymerase (RdRp). NSP15 encodes for RNA helicase. The structural genes encode the structural proteins, spike (S), envelope (E), membrane (M), and nucleocapsid (N), highlighted in green. The accessory proteins (shades of grey) are unique to SARS-CoV-2 in terms of number, genomic organization, sequence, and function (figure created with biorender.com).

The CoVs genome is a single-stranded positive-sense RNA (+ssRNA) molecule. The genome size ranges between 27–32 kbp, one of the largest known RNA viruses (Figure 1) [20,24]. The genomic structure of CoVs contains at least six open reading frames (ORFs). The first ORFs (ORF1a/b), located at the 5′ end, about two-thirds of the whole genome length, and encodes a polyprotein1a,b (pp1a, pp1b) [25]. Other ORFs are located on 3′ end encodes at least four structural proteins: envelop glycoprotein spike (S), responsible for recognizing host cell receptors [26]. Membrane (M) proteins, responsible for shaping the virions [27]. The envelope (E) proteins, responsible for virions assembly and release [28]. The nucleocapsid (N) proteins are involved in packaging the RNA genome and in the virions and play roles in pathogenicity as an interferon (IFN) inhibitor [29,30]. In addition to the four main structural proteins, there are structural and accessory proteins that are species-specific, such as HE protein, 3a/b protein, and 4a/b protein [24]. Once the viral genome enters the cytoplasm of the target cell, and given it is a positive-sense RNA genome, it translates into two polyproteins 1a, b (pp1a, pp1b). These polyproteins are processed into 16 non-structural proteins (NSPs) to form a replication-transcription complex (RTC) that is involved in genome transcription and replication. Consequently, a nested set of subgenomic RNAs (sgRNAs) is synthesized by RTC in the form of discontinuous transcription [24].

**Figure 2:**
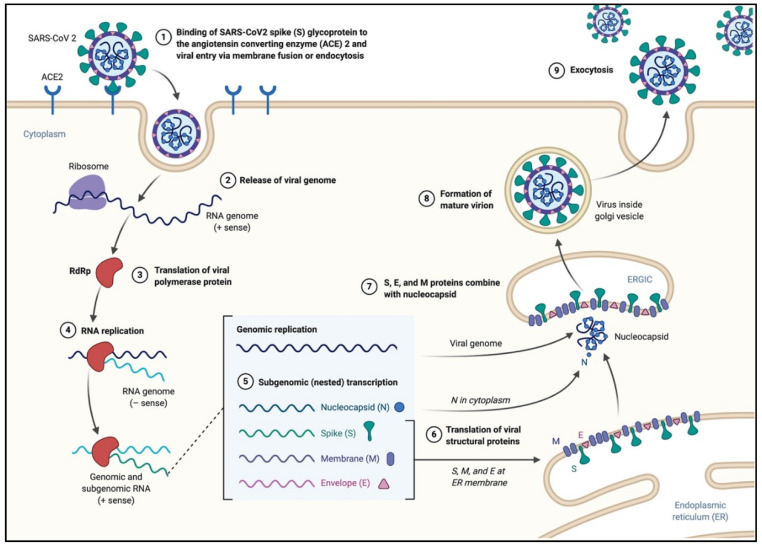
The life cycle of SARS-CoV-2 in the host cells. The S glycoproteins of the virion bind to the cellular receptor angiotensin-converting enzyme 2 (ACE2) and enters target cells through an endosomal pathway. Following the entry of the virus into the host cell, the viral RNA is unveiled in the cytoplasm. ORF1a and ORF1ab are translated to produce pp1a and pp1ab polyproteins, which are cleaved by the proteases of the RTC. During replication, RTC drives the production full length (−) RNA copies of the genome and used as templates for full-length (+) RNA genomes. During transcription, a nested set of sub-genomic RNAs (sgRNAs), is produced in a manner of discontinuous transcription (fragmented transcription). Even though these sgRNAs may have several open reading frames (ORFs), only the closest ORF (to the 5′ end) will be translated. Following the production of SARS-CoV-2 structural proteins, nucleocapsids are assembled in the cytoplasm and followed by budding into the lumen of the endoplasmic reticulum (ER)–Golgi intermediate compartment. Virions are then released from the infected cell through exocytosis (figure created with biorender.com).

SARS-CoV-2 primarily infects ciliated bronchial epithelial cells and type II pneumocytes, where it binds to the surface receptor, angiotensin-converting enzyme 2 (ACE2), through S glycoprotein found on its surface (Figure 2) [2,31,32,33]. When S glycoprotein binds to the ACE2, the cleavage of trimer S protein is triggered by the cell surface-associated transmembrane protease serine 2 (TMPRSS2) and cathepsin. S glycoprotein includes two subunits, S1 and S2. S1 determines the host range and cellular tropism and facilitates viral attachment to the target cells. S2 is a unit that mediates the fusion of viral and cellular membranes, ensuring viral entry through endocytosis. [31]. The affinity between the virus’s surface proteins and its receptors is a critical step for viral entry. Understanding the mechanism of SARS-CoV-2 could provide more insights into the viral transmission and reveal therapeutic targets. A recent study showed that the affinity between S glycoprotein of SARS-CoV-2 and ACE2 binding efficiency is 10–20 fold higher than that of SARS-CoV, which could explain the highly infectious ability of SARS-CoV-2 [4,34].

## 3. Potential COVID-19 Treatment

Antiviral drugs usually interfere with the viral replication machinery in the target cells. Given that viruses have similar actions of infection, different studies suggest the use of successful antiviral drugs for the treatment of COVID-19. Oseltamivir, peramivir, and zanamivir are neuraminidase inhibitors that have been used for influenza treatment. A recent report suggests the potential activity of these drugs against SAS-CoV-2 by interfering with the virions budding and releasing from the cell [35]. However, this virus uses different mechanisms other than neuraminidase to facilitate its release out of the infected cell. Another recent study suggests that ribavirin if combined with interferon-β, might be a promising drug for inhibition of SARS-CoV-2 replication [7]. Although this claim was based on previous in vitro findings [36], no other studies have shown the same results. Moreover, two reports did not recommend the use of this drug. On one hand, because it has no significant activity against SAS-CoV, and on the other hand, ribavirin is toxic with hemolysis activity [37,38]. Even though, there are many drugs with general antiviral activity; that can interfere with vial entry or blocking the virus receptors. Some examples are listed in Table 1. These drugs are either under investigation, showed antiviral activity against SARS-CoV-2, or constituted a treatment option of COVID-19.

### 3.1. Chloroquine and Hydroxychloroquine

Although many drugs have been reported to show promising results against SARS-CoV-2, none of these drugs have gained much attention like the antimalarial drugs Chloroquine (CQ) and Hydroxychloroquine (HCQ) [14,49,50]. HCQ is a CQ analog that has the same mechanism of action with a safer profile making it the prioritized drug to treat malaria, viruses, and autoimmune conditions [51]. There are several potential molecular mechanisms of HCQ action against SARS-CoV-2 that have been postulated. Generally, HCQ is a weak base drug that accumulates in the cell acidic compartments such as lysosomes and endosomes. This accumulation increases the pH and inhibits the maturation of these endosomal compartments. Therefore, HCQ might be interfering with the endocytosis of SARS-CoV-2 and subsequently affecting the virus entry and exit from the host cell [7,50]. Moreover, HCQ might reduce glycosylation of ACE2, interfering with SARS-CoV-2 to bind effectively to the host cell [51]. On the other hand, a recent publication hypothesizes that HCQ reduces the production of pro-inflammatory cytokines (such as interleukin-6, IFN-alpha, and TNF), thereby inhibiting various immune pathways that might lead to acute respiratory distress syndrome (ARDS) [52]. Currently, the use of hydroxychloroquine is approved by many of COVID-19 treatment protocols, especially in combination with other antibiotics and antiviral drugs, where others seriously considering it [1,4,40,50].

### 3.2. Remdesivir

Remdesivir is an adenosine nucleoside analog drug showing antiviral activity against Ebola and Marburg viruses, and other RNA viruses [53]. It acts through interfering with the viral RNA polymerase and evades proofreading by viral exoribonuclease, leading to a decrease in viral RNA production [54,55]. A recent report found that a combination of remdesivir and chloroquine inhibited SARS-CoV-2 in vitro [56]. Very recently, the drug gained more attention after a recent clinical trial found that remdesivir is highly effective in stopping the replication mechanism of SARS-CoV-2.

### 3.3. Losartan and Telmisartan

Losartan and telmisartan are drugs that belong to the angiotensin II receptor antagonist group. They block the substances that cause blood vessels narrowing, which lowers blood pressure and improves blood flow by relaxing the blood vessels. It has been suggested that Losartan can be used to reduce the aggressiveness and mortality of SARS-CoV-2 [57,58,59]. The fact that these drugs could block the ACE2 receptor should mean the ability to interfere with the entry of SARS-CoV-2 to the target cell. Using this drug group to treat COVID-19 seems vital at this point, particularly in combination with other antiviral drugs.

### 3.4. Baricitinib

Baricitinib is a drug used basically for the treatment of rheumatoid arthritis. It acts as an inhibitor for Janus kinase (JAK, U.S. Food and Drug Administration, 2018). Inhibition of the JAK pathway might interrupt the virus entry and the virions intracellular assembly by interfering with the endocytosis process [42].

### 3.5. Lopinavir/Ritonavir

It is a combination of both lopinavir and ritonavir drugs that have been used for HIV treatment and prevention. These drugs act by inhibiting the HIV protease enzyme through forming an inhibitor-enzyme complex [60]. A recent study showed that combination of these drugs reduced SARS-CoV-2 viral load and improved the clinical symptoms during the treatment of COVID-19 patients [43].

### 3.6. Darunavir

Darunavir is a second generation of HIV-1 protease inhibitors. It is a non-peptidic inhibitor of protease that enters itself in the active site of protease through several hydrogen bonds [61]. A recent report of an in vitro experiment indicated that using darunavir at a concentration of 300 μM, significantly inhibits SARS-CoV-2 replication. Its inhibition efficiency increased by 280-fold in comparison to the control group [44].

### 3.7. Camostat Mesylate

Camostat Mesylate is a type II transmembrane serine protease (TMSPSS2) inhibitor [62]. Since that SARS-CoV-2 entry mechanism involving the use of cellular protease TMPRSS2, a protease inhibitor would block entry and thus could be a treatment option [45].

### 3.8. Cepharanthie, Selamectin, and Mefloquine Hydrochloride

These are medications used as anti-malarial, antiviral, and anti-inflammatory activities. Those drugs showed significantly decreased viral RNA yield in treated cells, making them potential drugs for treating COVID-19 infections [47].

### 3.9. SARS-CoV-Specific Human Monoclonal Antibody (CR3022)

Passive immunization (PI) is a method to obtain instant, short-term fortification against infectious agents by introducing pathogen-specific antibodies to patients. These specific antibodies can bind to the pathogen antigens and block its interaction with a cellular receptor. It is extremely applicable in the case of viral antigens that facilitate the attachment to the target receptors [63]. After exposure to a viral infection, the body of the patient creates antibodies to fight off the virus. These antibodies in the blood of a recovered patient can be collected as convalescent plasma (CP) and transferred to the blood of a newly infected patient. Consequently, it can neutralize the pathogen, boost the immunity of that patient [64]. CR3022 is a human monoclonal antibody previously isolated from a convalescent SARS patient, used to target a highly conserved epitope that enables cross-reactive binding between SARS-CoV-2 and SARS-CoV [65]. CR3022 might have the potential to be candidate therapeutics for the prevention and treatment of COVID-19 patients, especially in life-threatening situations [66].

### 3.10. CRISPR/Cas13d System

Clustered regularly interspaced short palindromic repeats (CRISPR) is a family of repeated DNA sequences that can be found in the genomes of bacteria and archaea. These fragments are molecular remaining of bacteriophages infections and act as a defense mechanism to the prokaryotic cell. They can detect and destroy specific foreign DNA fragments, most probably from similar bacteriophages subsequent infections. CRISPR with chaperoning Cas proteins is a powerful technique that has been derived from the bacterial defense mechanism and implemented in gene editing of our interest [67]. Currently, there is an ambitious study suggesting that CRISPR/Cas13d system can be used to accurately digest the SARS-CoV-2 RNA genome, hence limiting its ability to reproduce [15]. Theoretically, this approach is excellent not only against COVID-19 but for the treatment and prevention of different RNA viruses’ infections. However, we have no idea if it is practical or not.

## 4. Conclusions

The COVID-19 pandemic poses a significant threat to the global public health systems. The increasing number of cases and deaths have put the international community on alert that the worst scenarios are possible. Despite our knowledge of the SARS-CoV-2 infectious cycle, there is no clear strategy for COVID-19 patients’ treatment. Based on recent experimental findings and recommendations, physicians are investigating different potential drugs that showed antiviral activity against SARS-CoV-2. Only time to tell which one of these drugs is going to work. In the meantime, scientists around the globe are working aggressively to find clinical therapies or vaccines against the virus.

## Figures and Tables

**Table 1:** Common and potent antiviral drugs.

Author	Drugs	Therapy Strategy Categories	Mechanisms of Therapy	Status
[14,39,40]	Chloroquine phosphate/ hydroxychloroquine	Anti-malaria anti-viral anti-inflammatory	Increasing endosomal pH, interfering with the glycosylation of cellular receptors of SARS-CoV-2, immunomodulator	FDA approved to be used in an emergency situation, implemented in many treatment protocols
[41]	Remdesivir	Antiviral drug (Nucleoside analogue)	Interfering with the viral replication	Investigational antiviral, clinical trials are in progress
[42]	Baricitinib	Rheumatoid arthritis (RA) drug, AP2-associated protein kinase 1 (AAK1) inhibitor	Interfering with viral entry by inhibiting one of the endocytosis regulators	FDA approved
[43]	lopinavir/ritonavir	HIV protease inhibitor	Could act by inhibiting SARS-CoV-2 protease for proteins cleavage, interfering with virus replication	FDA approved
[44]	Darunavir	HIV protease inhibitor	Could act by inhibiting SARS-CoV-2 protease for proteins cleavage, interfering with virus replication	FDA approved
[45]	Camostat Mesylate	Transmembrane protease, serine 2 (TMPRSS2) inhibitor	Interfering with viral entry	Japan approved
[46]	Favipiravir	Nucleoside analog	Binds to the viral RdRp and reduce its reproduction	Investigational
[47]	Cepharanthie, Selamectin, and mefloquine hydrochloride	Anti-viral Anti-inflammatory activities	Significantly reduced cytopathic effects of SARS-CoV-2, and decrease the viral load	Investigational
[48]	Ivermectin	Anti-parasite	Inhibits SARS-CoV-2 replication in vitro	FDA approved
